# News and Coming Events

**Published:** 1909-04-03

**Authors:** 


### News and Coming Events.

Sir Ernest F. G. Hatch has been unanimously elected Honorary Treasurer and Chairman of the Royal Ear Hospital, Dean Street, Sohc.

The Treasurers of the Middlesex Hospital have received a further donation of £200 to the General Fund from Lieutenant-Colonel H. J. Hope-Edwards.

The Duke of Argyll will preside at the 64th anniversary dinner of the German Hospital, Dalston, which will be held at the Whitehall Rooms on Friday, May 7.

A festival dinner in aid of the funds of the East London Hospital for Children, Shadwell, E., will be held at the Ritz Hotel on Wednesday, April 28. Field-Marshal Lord Grenfell will preside.

The annual general meeting of the Medical Sickness and Accident Society will be held on Thursday, May 27. Prospectuses and all further information can be obtained on application to Mr. F. Addiscott, secretary of the Society, 33 Chancery Lane, London, W.C.

A matinée in aid of the funds of the Army and Navy Male Nurses' Co-operation, a Society for the employment of duly qualified sailors and soldiers, to which we drew attention at its inception last year, will be held at The Playhouse, by permission of Mr. Cyril Maude, on June 22.

The Princess of Wales has given her patronage to the amateur theatrical matinée in aid of the Hospital for Women, Soho Square, and the East London Hospital for Children, Shadwell, which is being organised by Mrs. William D. James. Miss Lena Ashwell has kindly lent the Kingsway Theatre for the performance, which will take place on May 6. The play to be produced is "The Mollusc."

Princess Alexander of Teck will open the new Outpatient Department and Nurses' Home of the Royal National Orthopædic Hospital on Tuesday, April 20. The new buildings are in Great Portland Street, and the committee hope to arrange the opening of the new hospital itself during the coming summer. The treasurers, Lord Farquhar and Sir Richard Martin, have received a donation of £500 from the Goldsmiths' Company towards the reduction of the building fund debt of £25,000.

The Gilchrist Studentship for Women has been awarded by the Senate of University of London to Miss Enid Margaret Walters, M.B., B.S., London (Royal Free Hospital) School of Medicine for Women. The Studentship is of the value of £100, tenable for one year, and is open to graduates in Honours of the University, of not more than three years' standing from first graduation, who are prepared to take a course of study in an approved institution in preparation for some profession.

A White Paper has been issued by the Home Office containing tables of cases of industrial poisoning and accidents in factories and workshops in 1908. During the year 646 cases of lead poisoning were reported, and 32 deaths resulted from this cause. In addition to those included in the table 239 cases and 44 deaths were reported amongst house painters and plumbers. Ten cases of mercurial poisoning were reported and 23 of arsenical poisoning, as against seven and nine respectively during the previous year. Cases of anthrax numbered 47, as against 58 in 1907.

The next examination of candidates for commissions in the Royal Naval Medical Service will commence on May 10. The number of commissions to be granted will be fifteen. Further particulars will be supplied on application to the Medical Director-General, Admiralty, 18 Victoria Street.

The Prince of Wales has promised his patronage to a three days' bazaar and fête which the students of University College and Hospital are organising for the purpose of raising the £7,000 required to pay off the debt on their athletic ground and to make it their own freehold property. The Duke of Connaught will open the fête on the first day, July 1.

At an inquest held last week by Dr. Danford Thomas at Marylebone, on the body of a married woman, aged thirty-six years, the medical evidence showed that the cause of death was undoubtedly an overdose of quinine. The woman had taken 25 grains, and the post-mortem disclosed congestion of the brain, liver, and lungs. The other organs were healthy, and she was ordinarily a very healthy woman.

The Prince and Princess of Wales, Prince and Princess Christian of Schleswig-Holstein, the Duke and Duchess of Connaught, Princess Louise Duchess of Argyll, and Princess Henry of Battenberg have given their patronage to the annual festival ball in aid of the Italian Hospital, Queen Square, which will be held at the galleries of the Royal Institute of Painters in Water Colours, Piccadilly, on Monday, May 10.

The Duchess of Albany and Prince and Princess Alexander of Teck were present on March 19 at a musical and literary entertainment in aid of the National Hospital for the Paralysed and Epileptic held at 13 Carlton House Terrace, by permission of Sir Edwin and Lady Durning-Lawrence. Her Royal Highness, as President of the Jubilee Committee of the hospital, is now making a special appeal for £50,000 to celebrate its completion of 50 years of work. The entertainment attracted a large gathering, and the proceeds amounted to £244.

A report on the work of the Central Midwives Board has lately been issued as a White Paper. It deals with the period from the formation of the board in 1902 to March 31, 1908. On March 31, 1908, the number of names on the roll amounted to 23,634, but the report assumes that this figure is considerably in excess of the truth. It estimates the number of certified midwives in practice at present at 15,000. An important part of the board's work has been the exercise of its penal jurisdiction. In spite of the fact that the number of midwives reported by local supervising authorities for malpractice, negligence, or misconduct increases yearly it is believed that the board's administration of its penal powers has had a salutary effect on the conduct of the midwives in those districts in which the charges have arisen. The reports of the medical officers of health universally show the increasing value of the Midwives Act, though during the five years that it has been in operation various defects have appeared in its working. The most important of these are, first, the difficulty of collecting the contributions of the local supervising authorities; and, secondly, the omission to provide for the payment of the fees of medical practitioners summoned in emergencies on the advice of a midwife.

On March 24 the Prince of Wales received Sir William Church, Chairman of the Executive Committee, and Dr. E. F. Bashford, Director of the Imperial Cancer Research Fund, and considered the affairs and progress of the Fund, of which he is President.

The Medical Officer of Health for Bristol has issued a circular stating that of eight persons infected by a smallpox patient from outside the city on February 16, seven are now convalescent, and that there was only one notification last week. The outlook is most favourable, and no further cases are anticipated.

According to a Reuter's telegram from Berlin, dated March 26, Professor Greef, Director of the Berlin University Eye Hospital, claims that he has discovered the germ of trachoma, which, he says, is, in form, between a bacterium and a protozoon. As a result of the Professor's experiments with anthropoid apes, which he was enabled to carry out by means of a Government grant, he ascertained that trachoma is only contagious in the first stages.

The St. John Ambulance Association has received from Lord Rothschild, Chairman of the British Red Cross Society, two Diplomas of Honour, forwarded by Prince Hilkoff, Secretary of State to his Majesty the Emperor of Russia and President of the Russian Red Cross, awarded to the Association by the Committee of the Empress Marie Féodorovna Funds for the diminution of the sufferings of the sick and wounded in war. These Diplomas are awarded, one as a mark of approval of the litter on wheels exhibited at the eighth Red Cross Congress in London, 1907, and the other in recognition of the good work done by the Ambulance Department of the Order of the Hospital of St. John of Jerusalem in England for so many years past both in war and peace.

In aid of the Queen Alexandra Sanatorium at Davos an important matinée on a very large scale will be given on Tuesday, May 11, at Drury Lane Theatre. Mr. Arthur Collins has lent the theatre, and all the leading dramatic and variety artists have offered their services. The Queen, who is Patron of the institution, is taking a box, and on the suggestion of the President, Lord Balfour of Burleigh, the Council has agreed to place a bedroom at the disposal of the dramatic profession for every £500 realised by the matinée. The Sanatorium is designed for English-speaking persons suffering from pulmonary complaints who are able to pay a small fee but cannot afford hotel prices. It cost £36,000 to build, of which only £6,000 now remains unpaid.

The Education Committee of the London County Council report that a scheme for medical inspection has been submitted to the Board and approved by them. The Board do not insist upon their full requirements being carried out in respect of every child. The modified scheme provides that the complete inspection of every child admitted to or leaving school shall be in a limited number of schools, while in a further number of schools individual examination will be conducted, but the records will not be so elaborate as those prescribed by the regulations of the Board of Education. Individual examination will be made for the most part only when the children present abnormality. The committee propose that when the school doctor considers medical treatment necessary a notice shall be sent to the parents or guardians informing them of the nature of the child's affection and advising them to consult a doctor. They also propose that the parents and teachers shall be encouraged to attend the inspection of individual children.

The Therapeutical and Pharmacological section of the Royal Society of Medicine will meet at 20 Hanover Square, W., on Tuesday, April 6, at 4.30 p.m., when Dr. Gordon Sharp will read a paper on "Experiments and Experiences with the Heart Tonics—Pharmacological and Clinical," and Dr. William Bain on "The Action of the Digestive Ferments upon each other."

The Postmaster-General has decided that, in future, candidates for post-office employment who have conscientious objection to re-vaccination may be allowed exemption therefrom on making a statutory declaration to the effect that they conscientiously believe that re-vaccination would be prejudicial to their health. The Postmaster-General has refused, however, to grant exemption from primary vaccination.

The Rev. Walter Howse, M.A., of Campden Hill Court, W., late Mathematical Lecturer at King's College, London, left the residue of his property to Guy's Hospital, London, in augmentation of its endowment; and if by any chance this legacy shall lapse, it is to revert to Christ's College, Cambridge, at which College he graduated. It is estimated that the bequest to Guy's Hospital will amount to about £25,000.

Two inquests were held at Derby on March 26 on patients at the Derbyshire Royal Infirmary, who died whilst under anæsthesia. The coroner observed that it was a most unfortunate coincidence that two deaths should occur in such circumstances so close together, but there was no evidence of any blame attaching to the medical staff of the institution. In one case the patient was an old man of 76, admittedly in a moribund condition, suffering from blood poisoning, and an immediate operation was his only chance. The other patient was an infant.

The annual general meeting of the British Lying-in Hospital, Endell Street, W.C., was held on March 23. In proposing the adoption of the report and balance-sheet for 1908 the Chairman pointed out the hospital's progress since 1897, when seven beds were occupied daily, the number now being 22. This increase, and the appointment of a resident medical officer, has rendered most desirable the provision of more accommodation, which it is difficult to obtain and for which funds will be required. The accounts unfortunately showed a deficit of £673 for the year, and further public support is urgently needed.

The Hon. Sydney Holland presided on March 23 at the annual meeting of the Poplar Hospital for Accidents, and the report presented stated that the annual subscriptions amounted to £3,134 last year, against £3,180 in 1907, and donations to £4,845, compared with £3,212 in the previous year. In addition £1,285 was received for the building fund of the Convalescent Home. In-patients last year numbered 1,397, an increase of 26. Out-patients and casualties totalled 51,430, an increase of no fewer than 3,000 on the previous year. The total ordinary expenditure for the year amounted to £9,383, as against £9,256 in 1907. Reference was made to the intention of Mr. Holland to retire from the chairmanship. During the eighteen years that he has been identified with the hospital it has been almost entirely rebuilt, at a cost of £66,000, it has been trebled in size, and the reserve fund has been increased from £8,000 to £44,000. In response to the Committee's wishes he has agreed to remain as chairman with Mr. J. G. Broodbank as acting-chairman.

